# Supplementary material for: Is There a Universal Endurance Microbiota?
Source: Microorganisms. 2022 Nov 9;10(11):2213. doi: 10.3390/microorganisms10112213 (PMC9692344; doi:10.3390/microorganisms10112213)
Supplement: Supplementary file 1 [file microorganisms-10-02213-s001.zip › microorganisms-1958588-supplementary.pdf]

Is there a Universal Microbiota?

Olbricht et al.

microorganisms-1958588

Supporting Information

Table S1. Dataset characteristics for studies that were considered for reanalysis.
Table S2. Source of 16 target genera
Table S3. Simpson and Shannon diversity indices among studies and treatment groups therein.
Table S4. Normality, kurtosis and skewness of untransformed and square root transformed data
Table S5. Significant differences in relative abundance.
Table S6. Significant correlations
Table S7. Network descriptors for bacterial community associations
Table S8. Power analysis

Figure S1. Normality, kurtosis and skew for Boston Marathon dataset.
Figure S2. Normality, kurtosis and skew for Chinese Half Marathon dataset.
Figure S3. Normality, kurtosis and skew for USA professional cyclists dataset.
Figure S4. Relative abundances of target genera for Boston Marathon Athletes Before vs. After.
Figure S5. Relative abundances of target genera for Boston Marathon Controls vs. Athletes After.
Figure S6. Relative abundances of target genera for Chongqing International Half-Marathon Before vs. After.
Figure S7. Relative abundances of target genera for USA cyclists high vs. low training groups.
Figure S8. Network comparison for “athletes before” and “athletes after” the Boston Marathon.
Figure S9. Network comparison for sedentary controls to “athletes after” the Boston Marathon.
Figure S10. Network comparison for athletes before to athletes after the Chongqing International half-marathon.
Figure S11. Network comparison for the low training group of professional cyclists to the high training group of professional cyclists.

Supplemental Files (available in FigShare, DOI: 10.6084/m9.figshare.19799203)
Supplemental Fi1e 1. R script and input data to generate diversity index values and compare statistically.
Supplemental Fi1e 2. Bioinformatics pipeline (Geneious workflow)

Table S1. Dataset characteristics for studies that were considered for reanalysis. The first three criteria had to be satisfied as well as one or both of the last two criteria. Datasets chosen for reanalysis are indicated in bold.

**Reference**	**16S/ Illumina**	**Data Publicly Available**	**Endurance Event**	**Athletes Before v. After**	**Controls**
Allen et al. 2018 [1]	Yes	No	Yes	Yes	No
Bressa et al. 2017 [2]	Yes	Yes	No	No	Yes
Castellanos et al. 2020 [3]	Yes	Yes	No	No	Yes
Clarke et al. 2014 [4]	No	Yes	Yes	No	Yes
Craven et al. 2021 [5]	Yes	No	Yes	Yes	No
Grosicki et al. 2019[1] [6]	Yes	No	Yes	Yes	No
Jaago et al. 2021[2] [7]	Yes	No	Yes	Yes	No
Jang et al. 2019 [8]	Yes	No	Yes	No	Yes
Keohane et al. 2019 [9]	No	No	Yes	Yes	No
Kulecka et al. 2020 [10]	No	Yes	Yes	Yes	Yes
Munukka et al. 2018 [11]	Yes	Yes	No	Yes	No
Murtaza et al. 2019 [12]	Yes	No	Yes	Yes	No
**Petersen et al. 2017 [13]**	**Yes**	**Yes**	**No**	**No**	**No**
**Scheiman et al. 2019 [14]**	**Yes**	**Yes**	**Yes**	**Yes**	**Yes**

Tabone et al. 2021 [15]	Yes	No	Yes	Yes	No
Taniguchi et al. 2018 [16]	Yes	No	Yes	Yes	No
**Zhao et al. 2018 [17]**	**Yes**	**Yes**	**Yes**	**Yes**	**No**

[1]Raw data unavailable because sequencing company closed (Gregory Grosicki, personal communication)

[2]Raw data unavailable because authors only used reports from sequencing company (Kaia Palm, personal communication)

Table S2. Source of 16 target genera and statistical results from previous studies examined in this work.

**Study**	**Direction**	**Target taxa**	**Significance**
Scheiman et al. 2019 [14]	After > Before	Veillonella	Wilcoxon Rank Sum Test; P = 0.02
Zhao et al. 2018 [17]	Before > After	Bacteriodes_coprophilus	LEfSe Analysis; LDA score > 2; p < 0.05
		Clostridium_perfringens	
		Porphyromonadacae_bacterium	
		Phaseolus_vulgaris (aka Romboutsia)	
		Ezakiella	
		Prevotella_corporis	
		Clostridium_sp_YIT_12070	
	After > Before	Actinobacillus	LEfSe Analysis; LDA score < -2; p < 0.05
		Succinivibrionaceae	
		Ruminococcus_bicirulans	
		Ruminiclostridium_5	
		Mitsuokella	

		Collinsella_aerofaciens	
		Collinsella_aerofaciens	
		Coriobacteriaceae	
		Coriobacteriales	
		Coprococcus_2	
		Actinobacteria	
		Pseudobutyrivibrio	
Peterson et al. 2017 [13]	High volume > Low volume	Prevotella (cluster 1)	Fisher's exact test showing that cyclists who exercised >11 h/week were more likely to have ≥2.5% Prevotella; p-value= 0.0026; Approximate unbiased p-value of 94 for cluster 1
	Low volume > High volume	Bacteroides (cluster 2)	Approximate unbiased p-value of 90 for cluster 2
	"Mixed"	Eubacterium (cluster 3)	Approximate unbiased p-value of 76 for cluster 3
		Ruminococcus (cluster 3)	

		Akkermansia (cluster 3)	
		Methanobrevibacter smithii (from transcriptome comparisons)	More than 102x the number of transcripts detected compared to that expected from DNA sequencing; M. smithii gene expression was highly variable between cyclists but was highest in professional-level cyclists compared to CAT 1 cyclists as determined with Fisher’s exact test ($p < 0.001$).

Table S3. Simpson and Shannon diversity indices among studies and treatment groups therein. None of the comparisons between treatment groups were significantly different at alpha = 0.05.

Study	Treatment Group	Simpson D AVG (± SE)	Shannon AVG (± SE)
Marathon (Boston, USA)	Controls	0.814 (± 0.012)	1.702 (± 0.011)
	Athletes Before	0.815 (± 0.009)	1.675 (± 0.020)
	Athletes After	0.828 (± 0.011)	1.673 (± 0.024)
Half Marathon (Chongqing, China)	Athletes Before	0.885 (± 0.013)	2.871 (± 0.075)
	Athletes After	0.909 (± 0.006)	2.941 (± 0.045)
Cyclists (USA)	Low	0.819 (± 0.024)	2.288 (± 0.083)
	Medium	0.794 (± 0.018)	2.212 (± 0.061)
	High	0.743 (± 0.048)	2.125 (± 0.159)

Table S4. Normality testing for the data from three previously published studies with untransformed and square root transformed relative abundance data used in examining correlations.

Dataset	Number of Microbiota Samples	Number of Bacterial Genera[a]	Trans-formation	Percent Shapiro Wilks Tests p-value > 0.05[b]	Mean Kurtosis[c]	Mean Skewness[c]
Scheiman et al. [14]	40	57	None	0.0	24.79	3.87
Scheiman et al. [14]	40	57	Square-root	3.5	4.17	1.41
Zhao et al. [17]	38	79	None	2.5	8.02	2.57
Zhao et al. [17]	38	79	Square-root	15.2	2.48	1.36
Petersen et al. [13]	33	42	None	0.0	6.05	2.22
Petersen et al. [13]	33	42	Square-root	45.2	1.35	1.07

[a] pruned to only taxa present in >75% of samples for detecting correlations

[b] $p > 0.05$ indicates the distribution is not significantly different from normality

[c] Kurtosis and skewness values between -2 and +2 are consistent with a normal distribution

Table S5. Significant differences in relative abundance based on Wilcoxon tests for all genera (data exploration) from Boston Marathon “Athletes Before” vs. “Athletes After”, Boston Marathon Controls vs. “Athletes After”, Half Marathon “Athletes Before” vs. “Athletes After”, and Cyclists low vs. high training groups. Genera are separated by dataset, then sorted by increasing p-value.

	**Genus**	**P value**	**Mean Difference**[1]	**Mean Ratio**[2]
Boston Marathon “Athletes Before” vs. “Athletes After”	*Enterocloster*	0.015	<0.001	0.72
	Fournierella	0.017	<0.001	0.42
	Marvinbryantia	0.021	<0.001	16.47
	Clostridium+	0.041	0.003	3.60
Boston Marathon Controls vs. “Athletes After”	*Veillonella+*	0.002	0.003	22.48
	Alistipes	0.004	-0.042	0.39
	Ruthenibacterium	0.004	-0.002	0.12
	Butyricimonas	0.004	<0.001	0.12
	Phascolarctobacterium	0.007	-0.008	0.24
	Raoultella	0.008	-0.001	0.016
	Negativicoccus	0.016	<0.001	Inf
	Parasutterella	0.019	0.004	3.41
	Ihubacter	0.023	<0.001	0.33
	Christensenella	0.027	<0.001	0.20

	Negativibacillus	0.028	<0.001	0.23
	Oribacterium	0.029	<0.001	1.30
	Facklamia	0.031	<0.001	<0.01
	Gordonibacter	0.034	<0.001	0.50
	Citrobacter	0.035	-0.002	<0.01
	Cuneatibacter	0.041	<0.001	0.63
	Merdimonas	0.043	<0.001	0.31
	Anaeromassilibacillus	0.047	<0.001	0.56
Half Marathon "Athletes Before" vs. "Athletes After"	*Romboutsia**+	0.00021	-0.013	0.49
	Coprococcus+	0.00027	0.011	1.87
	Veillonella+	0.00042	<0.001	2.67
	Collinsella+	0.00052	0.004	1.95
	Tyzzerella	0.00052	-0.001	0.63
	Acidaminococcus	0.00064	<0.001	1.85
	Prevotellamassilia	0.00079	-0.006	0.38
	Ruminococcus+	0.0012	0.009	1.51
	Senegalimassilia	0.0021	<0.001	2.17
	Paeniclostridium	0.0024	<0.001	0.11

	Oxalobacter	0.0042	<0.001	0.40
	Barnesiella	0.0046	<0.001	0.69
	Mitsuokella+	0.0046	0.003	2.23
	Mediterranea	0.0059	-0.001	0.22
	Pediococcus	0.0059	<0.001	0.12
	Phascolarctobacterium	0.0062	0.002	2.12
	Butyrivibrio	0.0087	<0.001	2.50
	Roseburia	0.0094	0.016	1.30
	Streptococcus	0.0094	<0.001	1.18
	Peptacetobacter	0.0097	<0.001	0.40
	Terrisporobacter	0.010	<0.001	0.20
	Pyramidobacter	0.014	<0.001	0.18
	Ligilactobacillus	0.016	<0.001	1.33
	Butyricicoccus	0.017	-0.001	0.50
	Megamonas	0.018	0.008	1.55
	Citrobacter	0.021	<0.001	0.15
	Slackia	0.021	<0.001	1.72
	Eubacterium+	0.026	0.002	1.16

	Faecalitalea	0.029	<0.001	4.88
	Pseudoflavonifractor	0.029	<0.001	1.31
	Herbinix	0.036	<0.001	8.59
	Akkermansia+	0.038	<0.001	0.28
	Eisenbergiella	0.042	<0.001	0.38
	Olsenella	0.044	<0.001	3.01
	Shigella	0.044	<0.001	0.50
	Parasporobacterium	0.045	<0.001	1.68
Cyclists Low Training Group vs. High Training Group	*Prevotella*+*	0.00031	0.232	700.38
	Romboutsia+	0.0047	0.003	8.28
	Turicibacter	0.0047	<0.001	5.77
	Bacteroides+	0.0070	-0.065	0.39
	Parabacteroides	0.015	-0.026	0.21
	Pseudoflavonifractor	0.021	-0.001	0.23
	Flavonifractor	0.031	<-0.001	0.45
	Massiliprevotella	0.032	0.002	Inf
	Phascolarctobacterium	0.035	-0.004	0.02
	Alistipes	0.038	-0.005	0.76

	Dorea	0.038	0.004	3.69
	Faecalibacterium	0.038	-0.074	0.48
	Faecalicatena	0.050	<-0.001	0.66
	Holdemania	0.050	<-0.001	0.11

[1] Mean Difference calculated as After - Before (Boston Marathon); After - Control (Boston Marathon); After - Before (Half Marathon); High - Low (Cyclists)
[2] Mean Ratio calculated as After/Before (Boston Marathon); After/Control (Boston Marathon); After/Before (Half Marathon); High/Low (Cyclists)
*Significant after BH correction (BH corrected alpha value: Boston Marathon Athletes Before vs. After = 0.000177, Boston Marathon Athletes vs. Controls = 0.000177, Half Marathon = 0.00025, Cyclists low vs. high = 0.00033)
+One of the 16 target genera identified from previously published results

Table S6. Significant Spearman correlations on all bacterial genera present in >75% of samples that are significantly correlated in the endurance group compared to the non-endurance group using relative abundances after Benjamini and Hochberg (BH) correction

Dataset	Treatment Group Comparison	Genus 1	Genus 2	BH Corrected Spearman p-value[a]
Boston Marathon	Before vs. after	None		
	Controls vs. after	None		
Half Marathon	Before vs. after	Bacteroides	Prevotella	0.0063
		Erysipelatoclostridium	Megamonas	0.0063
		Alistipes	Faecalimonas	0.0063
		Bacteroides	Megamonas	0.0063
		Bacteroides	Erysipelatoclostridium	0.0108
		Bacteroides	Fusicatenibacter	0.0140
		Parasutterella	Ruthenibacterium	0.0178
		Butyricicoccus	Prevotellamassilia	0.0181
		Fusicatenibacter	Megamonas	0.0181
		Flavonifractor	Paraprevotella	0.0222
		Parasutterella	Prevotella	0.0222

		Enterocloster	Prevotella	0.0222
		Bacteroides	Klebsiella	0.0233
		Haemophilus	Megasphaera	0.0233
		Alistipes	Butyricimonas	0.0247
		Catenibacterium	Mitsuokella	0.0250
		Fusicatenibacter	Prevotella	0.0250
		Holdemanella	Intestinibacter	0.0371
		Lachnobacterium	Murimonas	0.0384
		Holdemanella	Sutterella	0.0384
		Bacteroides	Parasutterella	0.0420
		Erysipelatoclostridium	Fusicatenibacter	0.0420
		Acidaminococcus	Anaerobutyricum	0.0420
		Anaerostipes	Fusicatenibacter	0.0420
		Blautia	Fusicatenibacter	0.0420
		Lachnospira	Lactobacillus	0.0420
		Enterocloster	Parasutterella	0.0428

		Dialister	Oscillibacter	0.0495
Professional Cyclists	Low vs. High Training Group	None		

[a] Benjamini and Hochberg (BH) corrected p-values at alpha = 0.05 (Scheiman et al. n tests = 1596; Zhao et al. n tests = 3081; Petersen et al. n tests = 861)

Table S7. Network descriptors for bacterial community associations among the top 100 bacterial genera in each treatment group for the three datasets. LCC = largest connected component; Dissimilarity = 1 - edge weight

Dataset	Group	N Samples	Number of Nodes in the LCC	Percent Positive Edges in LCC	Average Dissimilarity in the LCC	Average Path Length in the LCC
Boston Marathon	Controls	82[a]	55	73.3	0.9759	2.53
	Athletes Before	55[b]	24	90.0	0.9223	1.82
	Athletes After	63	37	80.0	0.9594	2.71
Half Marathon	Before	19	27	73.7	0.9459	1.64
	After	19	38	82.5	0.9364	1.65
Cyclists	Low	8	42	75	0.9463	1.80
	High	8	41	67.5	0.9516	1.65

[a] Two samples with <1000 reads removed (SG29.C.D+1 and SG30.C.Day+3)
[b] Two samples with <1000 reads removed (SB01.AB.Day-5 and SB12.AB.Day-5)

Although there are no consistent changes in the networks across datasets, below is a brief summary of changes in pairwise bacterial associations noted within each individual dataset.

Boston Marathon study

Network analysis was used to identify clusters of bacterial genera that may be associated with each other (beyond simple pairwise correlations). Controls and "athletes after" had clusters ranging from 0 to 11 genera with the most common clusters containing three and four genera, respectively. "Athletes after" had clusters ranging from zero to 12 with the most frequent clusters

containing five genera. The relative LCC for athletes after was 54.2% higher than "athletes before" and 32.7% lower than controls (Supplemental Table 6). The highest percentage of positive associations was in the "athletes before" network (90%) and the lowest was in the control network (73.3%). "Athletes before" had the lowest average dissimilarity value (0.9223) and lowest average path length (1.82). “Athletes after” had the highest average path length (2.71), yet the controls had the highest average dissimilarity value (0.9759) (Supplemental Table 6).

In comparing two treatment groups at a time, we started with "athletes before" vs "athletes after" (Supplemental Figure 5). Following a filtering step for taxa and samples, we were left with 93 bacterial genera. For the Jaccard analysis of the multiple centrality measures, only the number of hub taxa was significantly different between "athletes before" vs. "athletes after" (p = 0.017; Jaccard index = 0 indicating no overlap in the hub taxa between the two networks). An adjusted Rand index of 0.507 (p = 0 meaning significantly different from zero) indicates significant differences between the clusters in the two networks. The top five genera whose number of edges changed the most were all higher in "athletes after" and lower in "athletes before" (Supplemental Figure 5). For example, *Veillonella* ties for second with *Succinivibrio*, both having four positive associations in athletes after and only one for "athletes before". However, none of the three centrality metrics (degree, betweenness, closeness) were significantly different for any genera across the networks after permutation testing and adjusting significance using the local false discovery rate.

In our comparisons of controls versus "athletes after", after filtering, there were 84 bacterial taxa remaining (Supplemental Figure 6). For the Jaccard index analysis of centrality measures, only the number of hub taxa was significantly different (p = 0.017; Jaccard index = 0 indicating no overlap in the hub taxa between the two networks). Betweenness was close to significant (betweenness value = 0.200, 1000 permutations, p = 0.063). An adjusted Rand index of 0.121 (p = 0 meaning significantly different from zero) indicates the clusters in the two networks are different. The top five genera whose number of edges changed the most were all higher in controls and lower in "athletes after". For example, *Faecalibacterium* has nine associations in controls and only two in "athletes after". *Veillonella* ranks 10th and has four associations after the marathon and none in the controls. Although the aforementioned are among the most extreme, permutation testing (n = 1000) indicates the following genera are significantly different in their degree of association (number of edges; p = 0.044): *Dialister, Erysipelatoclostridium, Terrisporobacter, Lachnospira, Phascolarctobacterium, Barnesiella, Lactobacillus, Merdimonas, Coprococcus, Parasutterella, Coprobacter,* and *Sutterella* (Figure 8). None of the other two network centrality metrics (betweenness and closeness) showed any significant results for any taxa.

Chinese Half Marathon study
For the Zhao et al. datatset, the 398 genera were filtered to the top 100 most common taxa. The network based on the before group is much sparser (27 nodes) than the network for the after group (38 nodes) (Supplemental Table 6, Supplemental Figure 7). There were 8.8% more positive edges in the after group than in the before group. Clusters contained up to 10 taxa in the before group (the most frequent being five taxon clusters) compared to a maximum of seven

taxon clusters in the after group (the most frequent being three taxon clusters) (Supplemental Table 6).

In comparing the before and after groups, *Blautia* and *Fusicatenibacter* are hubs in both networks (Supplemental Figure 7). In the network based on the before group data, *Coprococcus* has the highest number of edges (7) and there is a three way tie among *Fusicatenibacter, Anaerostipes* and *Faecalimonas* for the second most "edgy" bacteria with six connections each. In the "athletes after" network, *Bacteroides* has the highest number of edges (12), while *Blautia* and *Anaerobutyricum* are second with 11 edges each (Supplemental Figure 7). All network descriptors are not significant, but the Jaccard distances for "closeness" is nearly so (P = 0.0589). The Rand index (ARI = 0.219) is significantly different from zero (1000 permutations, p = 0) indicating we can reject the null hypothesis that the two networks are completely different random clusterings. Among the top 50 taxa in the union network, there were ~2x more genera that increased their connections in the after group compared to before (32 increases, 15 decreases).

Cyclist Training Groups study

For the Petersen et al. dataset we focused on comparing the low vs. high training groups (174 bacterial genera, no samples filtered). No network was produced using the t-test criteria with p-value of 0.2, so we applied a raw threshold cutoff (L = 0.7 for low and 0.75 for high; Supplemental Table 6). The individual networks based on the low and high training groups are very similar in their number of nodes (40 vs. 41 nodes respectively) and positive edge percentage (75 vs. 67.5%; Supplemental Table 6). Clusters in the low training group contained up to twelve taxa with the most common being five taxa clusters and twelve taxa clusters (both have n = 6). Clusters in the high training group contained up to twelve taxa with the most common being ten taxa clusters (n=11).

The comparison between low and high training groups had 81 bacterial taxa (Supplemental Figure 8). *Anaerobutyricum* was a hub in the networks of both training groups, but the low training group also had *Anaerostipes, Blautia,* and *Enterocloster* as hubs while the high training group had *Dorea, Romboutsia*, and *Roseburia* as hubs. In the low training group, *Anaerostipes* had the highest number of edges (10) whereas in the high training group, *Phocaeicola* had 10 edges and *Roseburia* had nine edges (Supplemental Figure 8).

Table S8. Power analysis of four treatment group comparisons reanalyzed herein to determine the recommended sample size.

Event	Treatment Group Comparison	Published Sample Size	Genera Detected	Mean d (Max d)	Bonferroni Corrected Alpha	Recommended N Based on Average d[1] (N Based on Max d)
Boston Marathon	Before vs. After	15	282	0.0872 (0.247)	0.000177	2781 (353)
Boston Marathon	Athletes vs. Controls	15 vs. 10	282	0.0827 (0.212)	0.000177	6179 (941)
Chongqing International Half Marathon	Before vs. After	20[2]	198	0.0819 (0.273)	0.000253	3030 (279)
Competitive Cyclists	Low vs. High	8 vs. 8	148	0.0899 (0.171)	0.000337	4857 (1339)

[1] Recommended sample size calculated using power of 0.8, empirically determined d averaged across genera assuming 10% difference in mean abundance, and significance level of 0.05 with a Bonferroni correction for the number of tests based on the number of genera detected. Recommended sample sizes are reported as the number of pairs (Before vs. After) or number of samples in *each* treatment group (Athletes, Controls, Low, or High).
[2] Although the published sample size was 20, the empirically determined d is based on 19 paired samples since one sample could not be used.

Fig S1.Boston

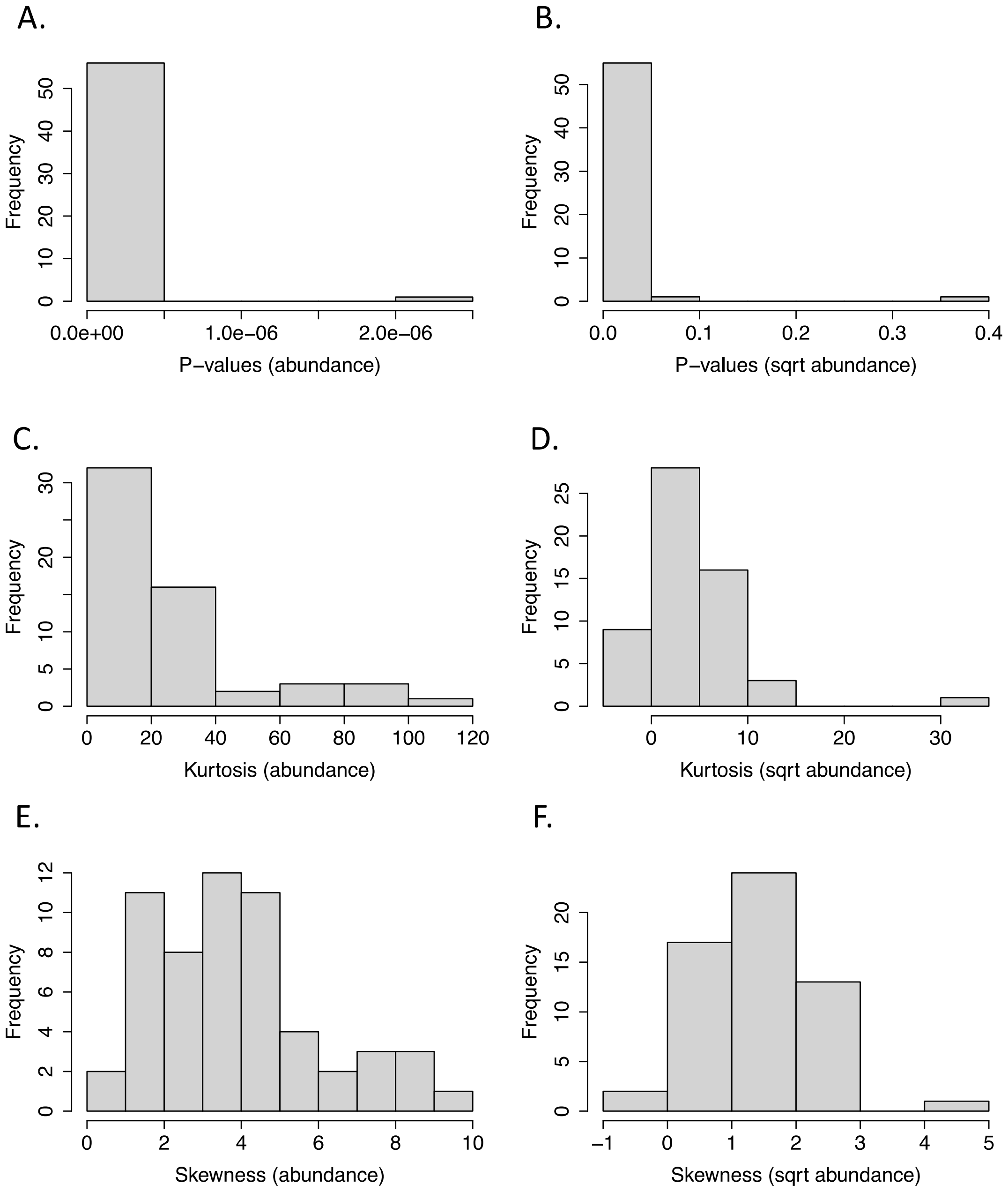

Fi[illegible]
tra[illegible]
B)[illegible] ls
are[illegible]
abundance values (B, D, F).

Fig S2.Half Marathon

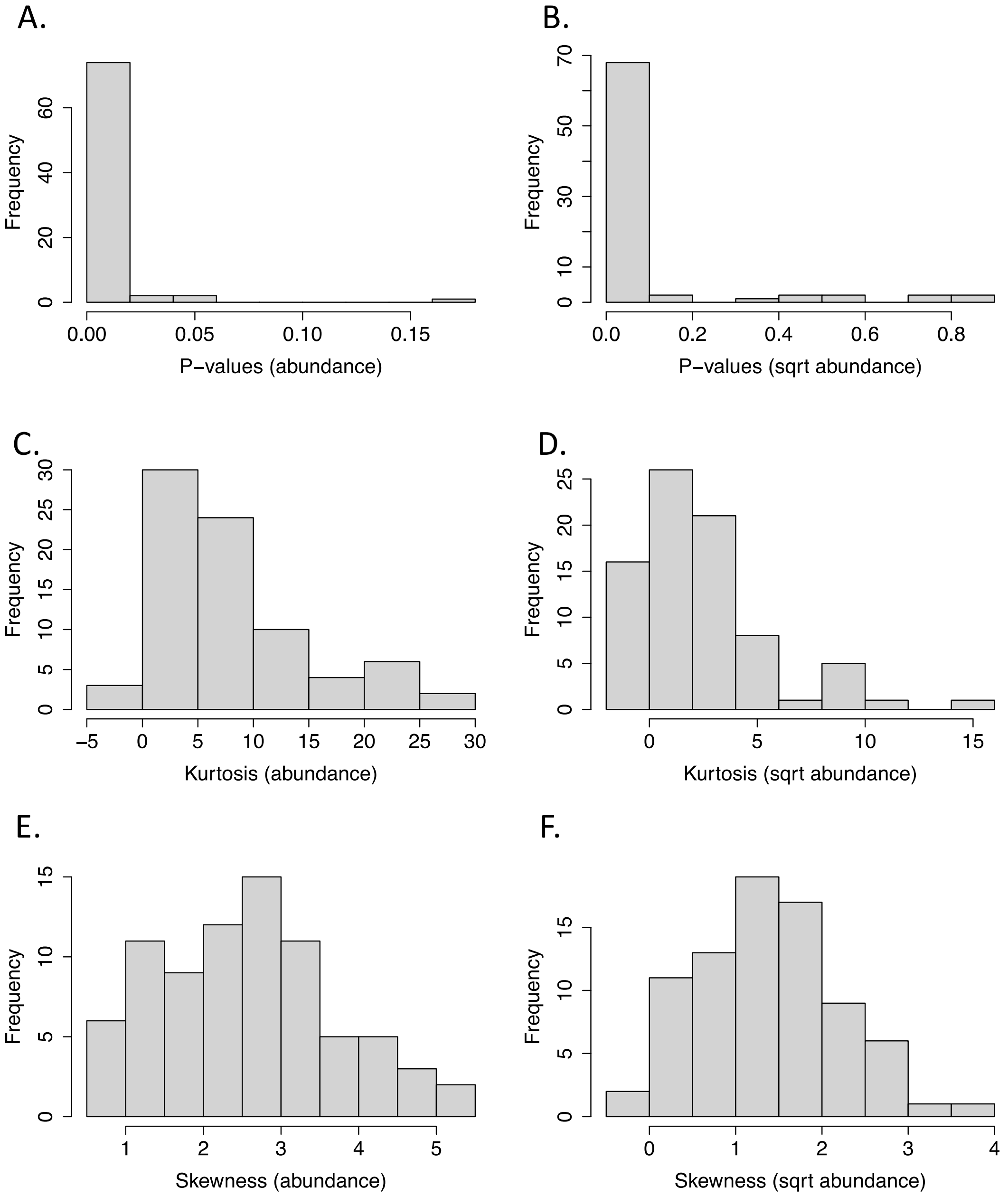

Fi[illegible]
tra [illegible] ts
(A [illegible]
pa [illegible]
ab [illegible]

Fig S3.Cyclists

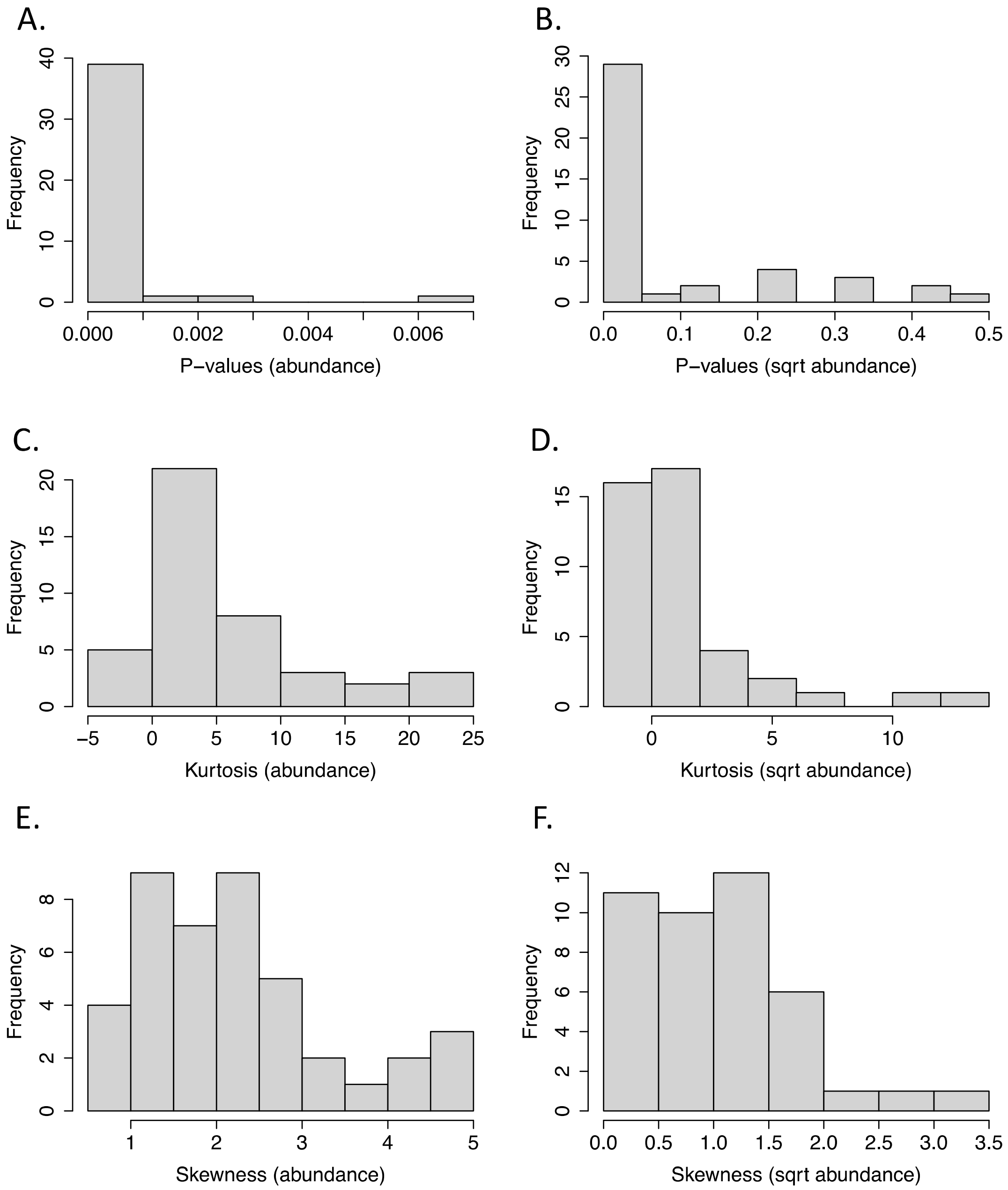

Fi[illegible]
tra[illegible]
tes[illegible]
Le[illegible]
transformed abundance values (B, D, F).

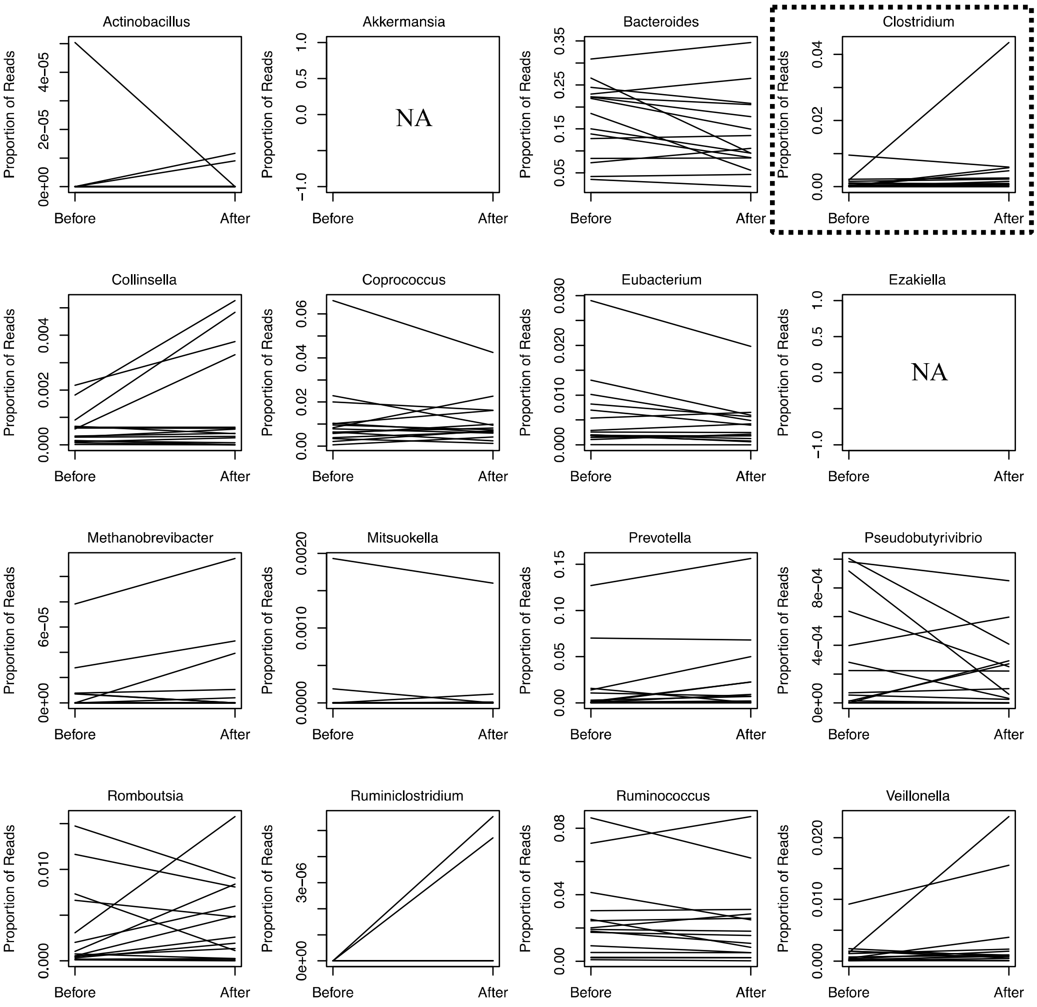

Scheiman B vs A

Fi [illegible] s
aft [illegible]
Si [illegible]
the [illegible]
inc [illegible]

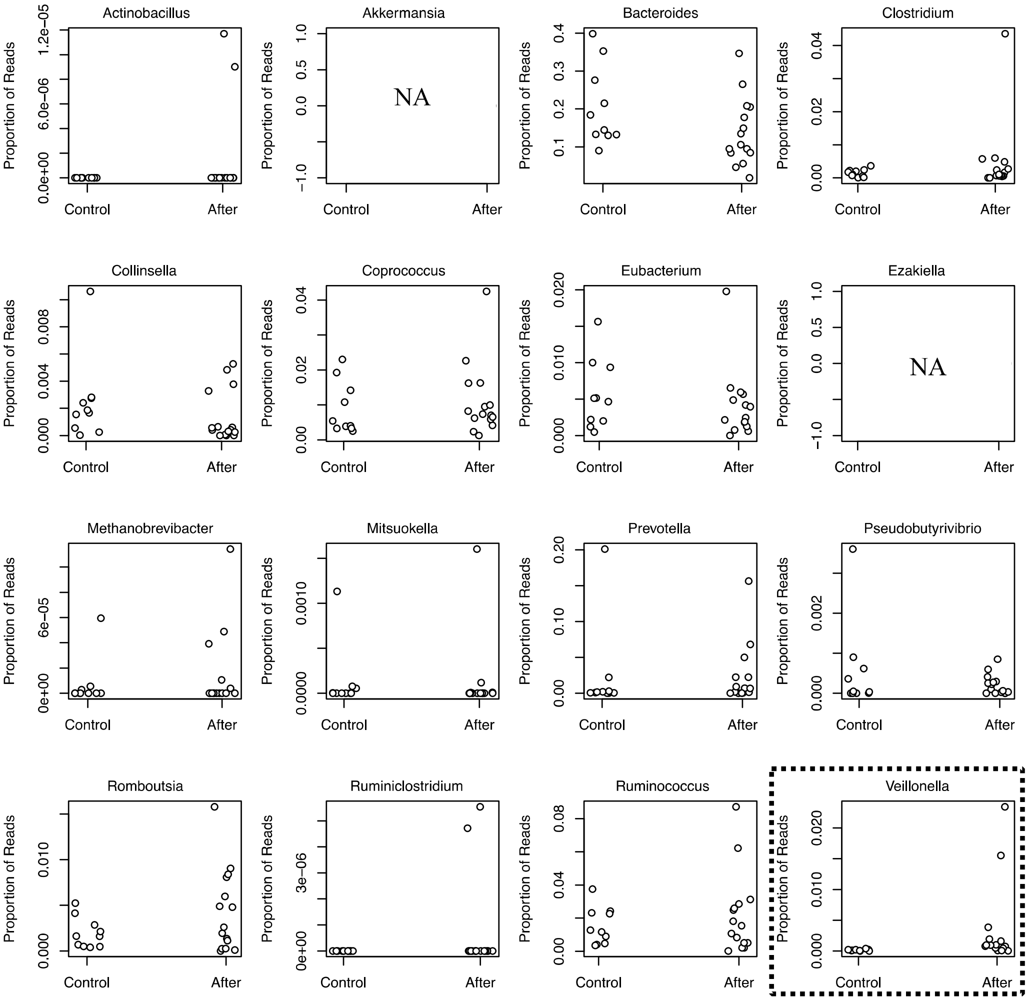

Scheiman C vs A

Fi
aft
Si
the
vis

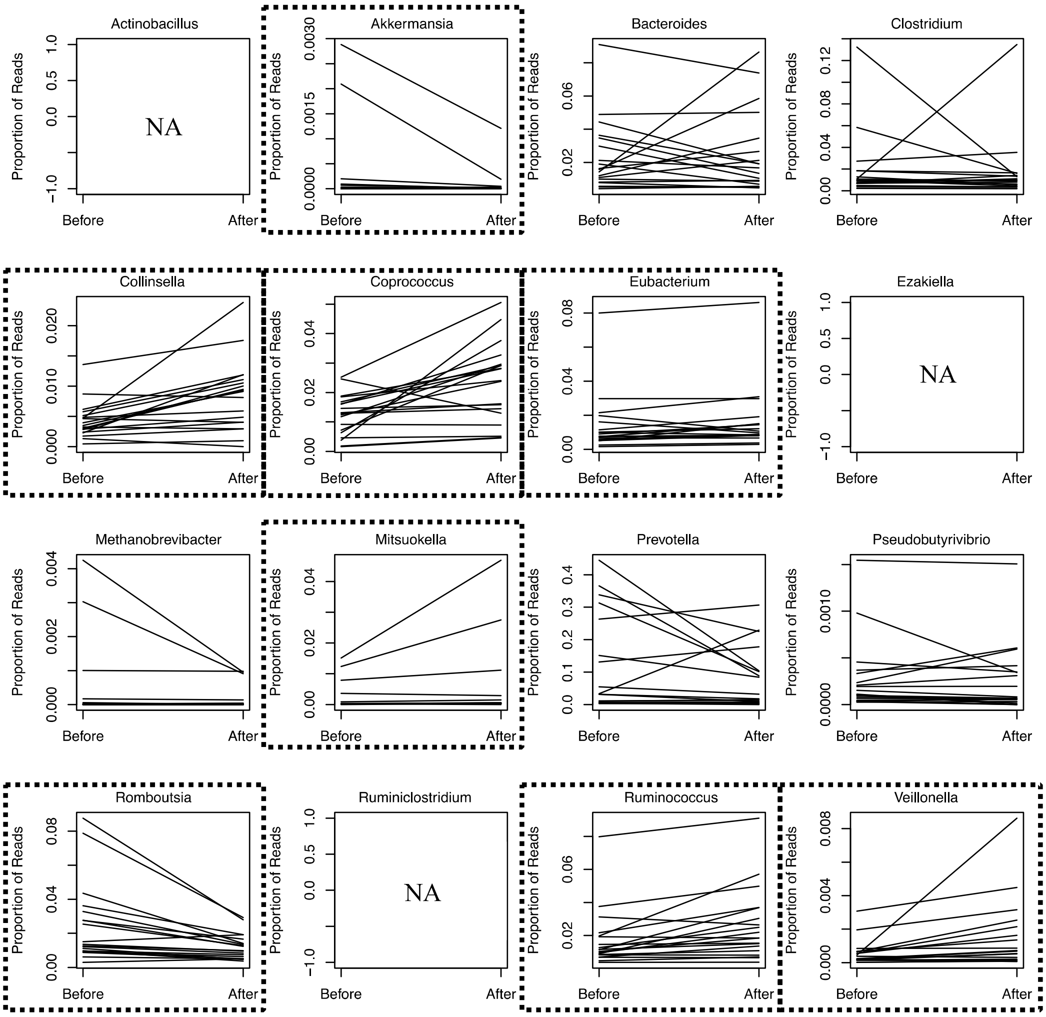

Zhao B vs. A

Fi [illegible] es
aft [illegible]
Si [illegible]
the [illegible] vo
tin [illegible]

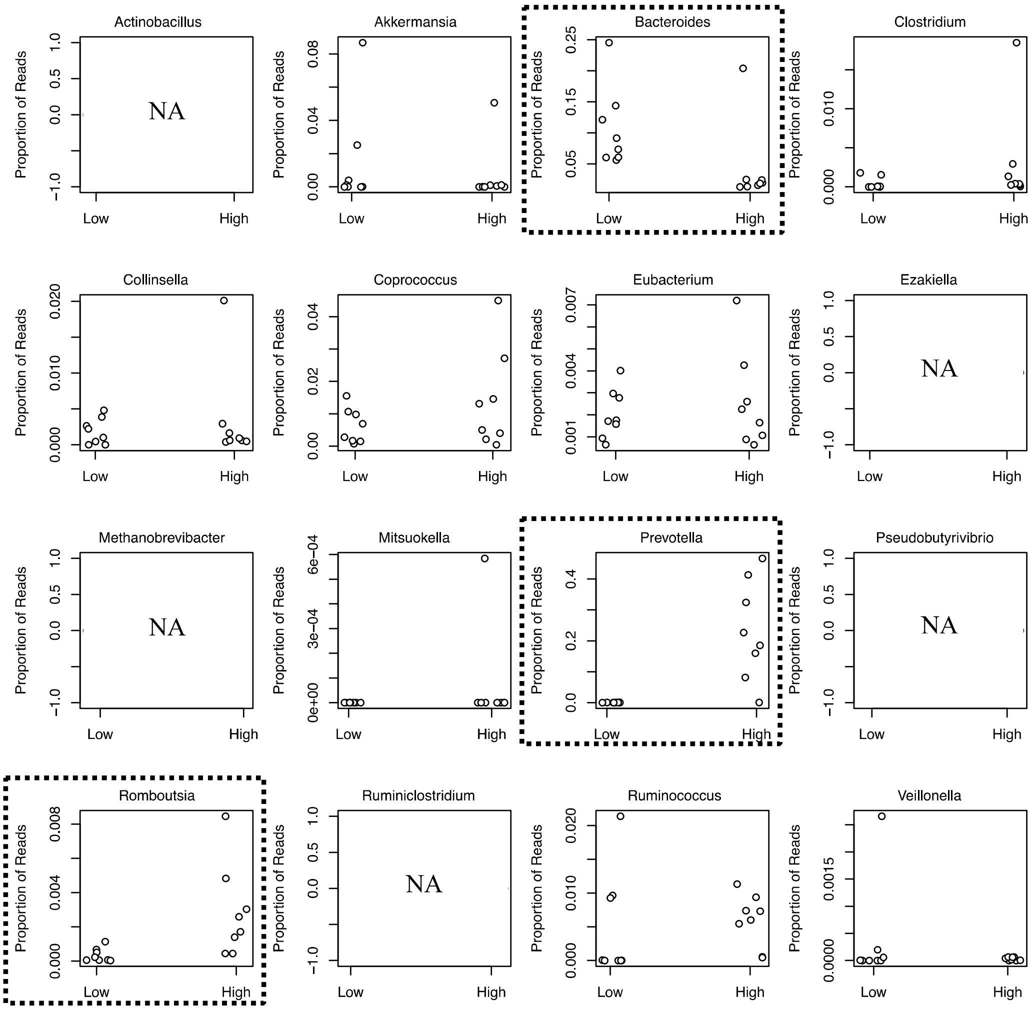

Petersen L vs. H

Fi[illegible] [illegible] g
gr[illegible] [illegible] :.
Si[illegible]
the[illegible]
vis[illegible]

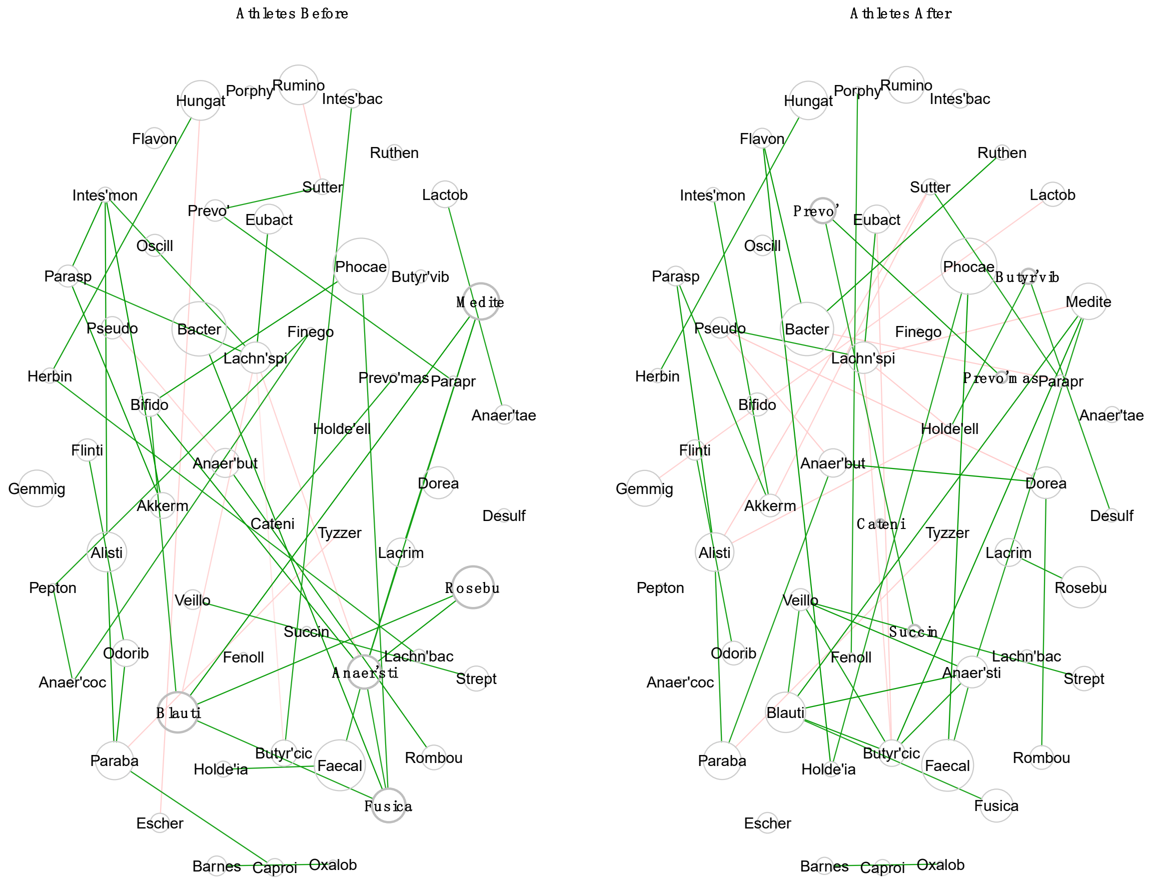

Figure S8. Network comparison for the Boston Marathon dataset comparing "athletes before" and "athletes after" the Boston Marathon. Nodes are bacterial genera. Node colors indicate clusters, line colors indicate positive associations (green) and negative associations (red), line weights reflect eigenvalues (connectedness). Line lengths are arbitrary. Hubs (bold font) are nodes with an eigenvector centrality above the empirical 95% quantile of all eigenvector centrality values.

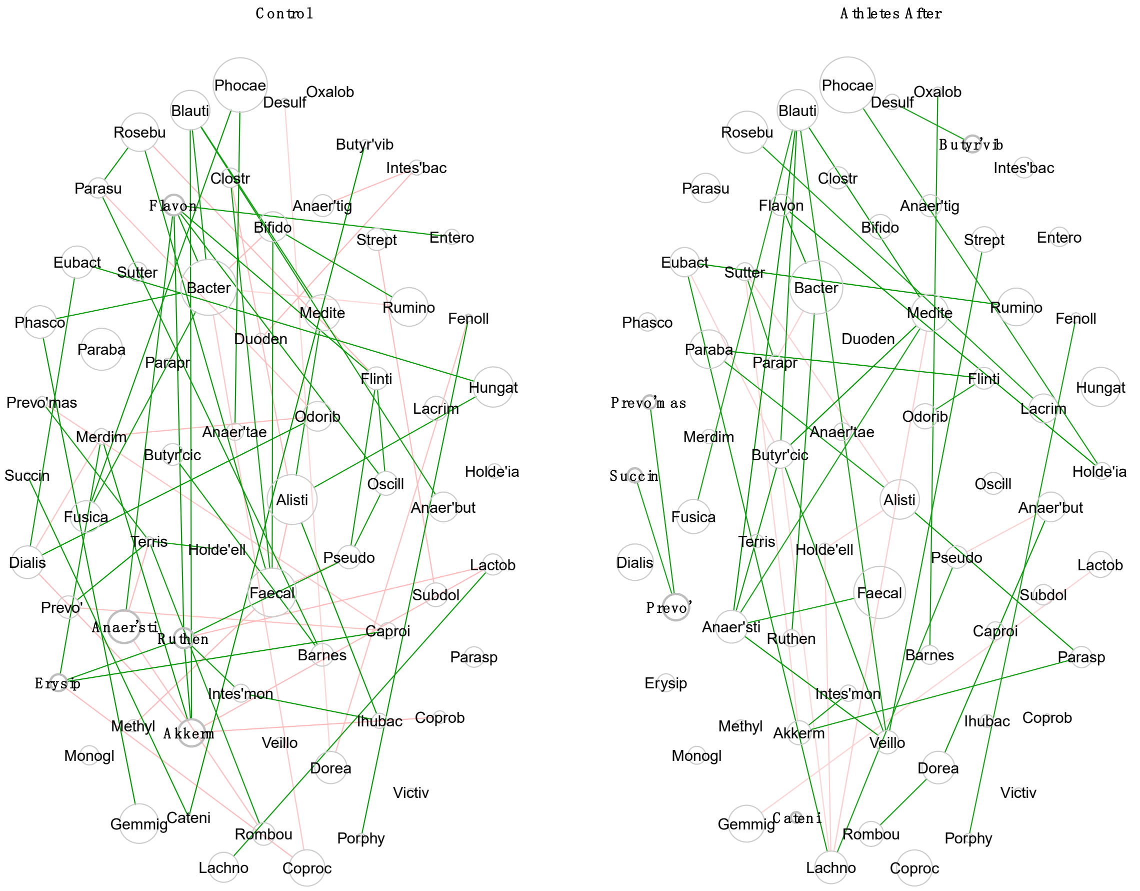

Figure S9. Network comparison for the Boston Marathon dataset comparing sedentary controls to “athletes after” the Boston Marathon. Nodes are bacterial genera. Node colors indicate clusters, line colors indicate positive associations (green) and negative associations (red), line weights reflect eigenvalues (connectedness). Line lengths are arbitrary. Hubs (bold font) are nodes with an eigenvector centrality above the empirical 95% quantile of all eigenvector centrality values.

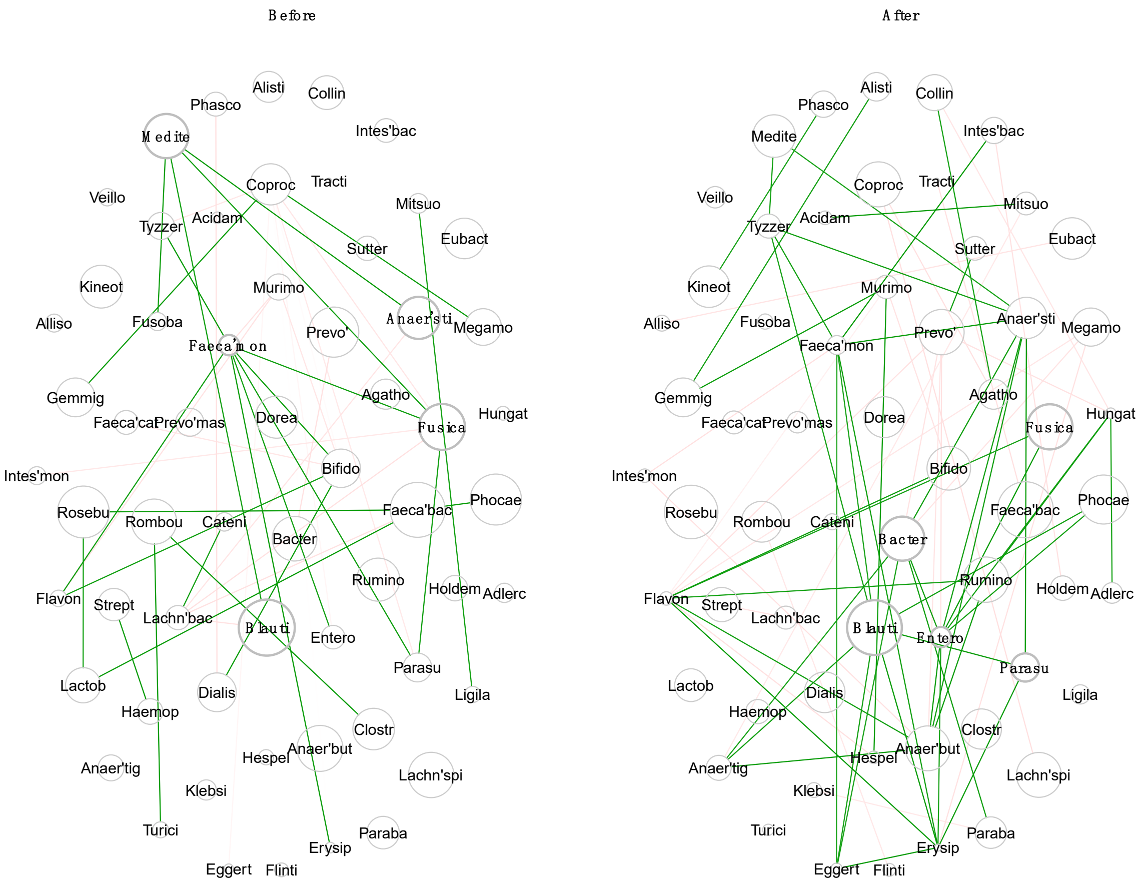

Figure S10. Network comparison for the Half Marathon dataset comparing athletes before to athletes after the Chongqing International half-marathon. Nodes are bacterial genera. Node colors indicate clusters, line colors indicate positive associations (green) and negative associations (red), line weights reflect eigenvalues (connectedness). Line lengths are arbitrary. Hubs (bold font) are nodes with an eigenvector centrality above the empirical 95% quantile of all eigenvector centrality values.

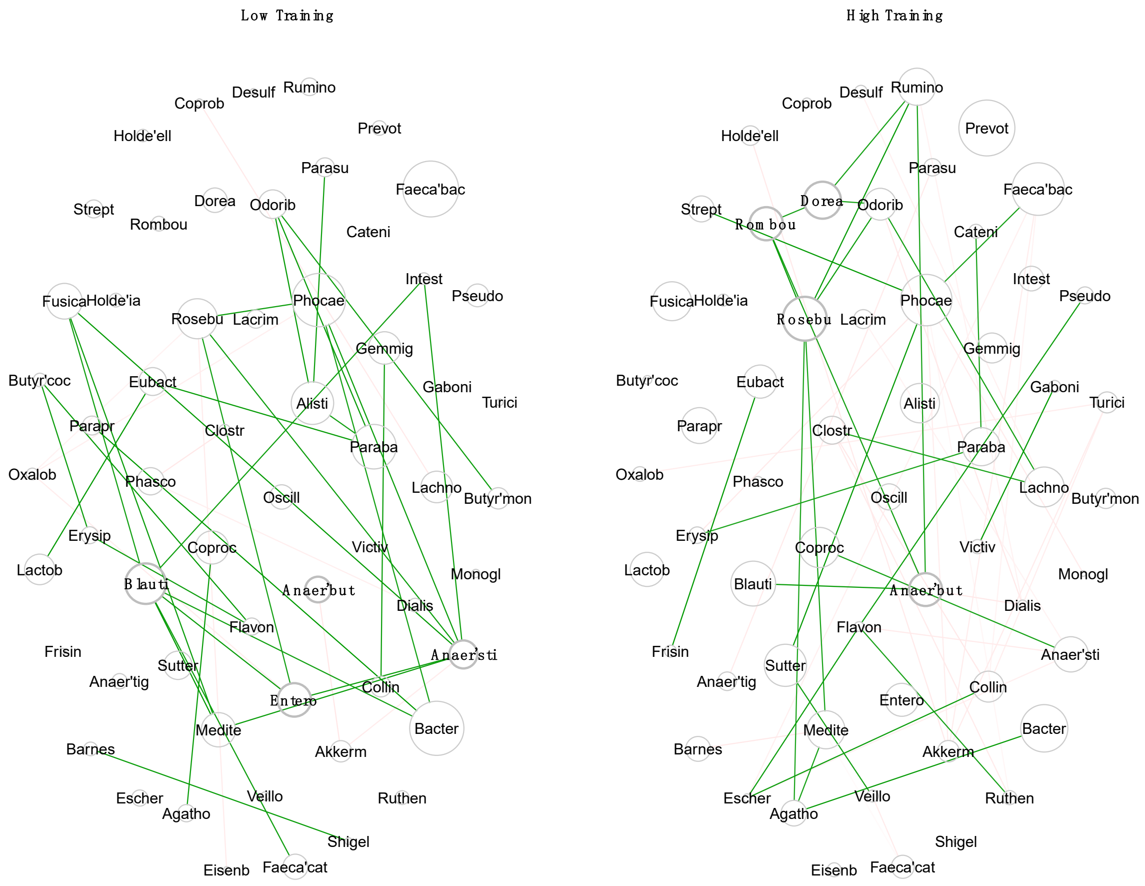

Figure S11. Network comparison for professional cyclists dataset comparing the low training group of professional cyclists to the high training group of professional cyclists. Nodes are bacterial genera. Node colors indicate clusters, line colors indicate positive associations (green) and negative associations (red), line weights reflect eigenvalues (connectedness). Line lengths are arbitrary. Hubs (bold font) are nodes with an eigenvector centrality above the empirical 95% quantile of all eigenvector centrality values.

References

1. Allen JM, Mailing LJ, Niemiro GM, Moore R, Cook MD, White BA, et al. Exercise Alters Gut Microbiota Composition and Function in Lean and Obese Humans. Med Sci Sports Exerc. 2018 Apr;50(4):747-757.

2. Bressa C, Bailén-Andrino M, Pérez-Santiago J, González-Soltero R, Pérez M, Montalvo-Lominchar MG, et al. Differences in gut microbiota profile between women with active lifestyle and sedentary women. PLoS One. 2017 Feb 10;12(2):e0171352.

3. Castellanos N, Diez GG, Antúnez-Almagro C, Bailén M, Bressa C, González Soltero R, Pérez M, Larrosa M. A critical mutualism–competition interplay underlies the loss of microbial diversity in sedentary lifestyle. Frontiers in microbiology. 2020 Jan 22;10:3142.

4. Clarke SF, Murphy EF, O'Sullivan O, Lucey AJ, Humphreys M, Hogan A, et al. Exercise and associated dietary extremes impact on gut microbial diversity. Gut 63.12 (2014): 1913-1920.

5. Craven J, Cox AJ, Bellinger P, Desbrow B, Irwin C, Buchan J, et al. The influence of exercise training volume alterations on the gut microbiome in highly-trained middle-distance runners. Eur J Sport Sci. 2021 Jun 13:1-9.

6. Grosicki GJ, Durk RP, Bagley JR, Rapid gut microbiome changes in a world-class ultramarathon runner. Physiological reports 7.24 (2019): e14313.

7. Jaago M, Timmusk US, Timmusk T, Palm K. Drastic Effects on the Microbiome of a Young Rower Engaged in High-Endurance Exercise After a Month Usage of a Dietary Fiber Supplement. Frontiers in Nutrition 8 (2021).

8. Jang LG, Choi G, Kim SW, Kim BY, Lee S, Park H. The combination of sport and sport-specific diet is associated with characteristics of gut microbiota: an observational study. J Int Soc Sports Nutr. 2019 May 3;16(1):21.

9. Keohane DM, Woods T, O'Connor P, Underwood S, Cronin O, Whiston R, et al. Four men in a boat: Ultra-endurance exercise alters the gut microbiome. Journal of science and medicine in sport 22.9 (2019): 1059-1064.

10. Kulecka M, Fraczek B, Mikula M, Zeber-Lubecka N, Karczmarski J, Paziewska A, et al. The composition and richness of the gut microbiota differentiate the top Polish endurance athletes from sedentary controls. Gut microbes 11.5 (2020): 1374-1384.

11. Munukka E, Ahtiainen JP, Puigbó P, Jalkanen S, Pahkala K, Keskitalo A, et al. Six-Week Endurance Exercise Alters Gut Metagenome That Is not Reflected in Systemic Metabolism in Overweight Women. Front Microbiol. 2018 Oct 3;9:2323.

12. Murtaza N, Burke LM, Vlahovich N, Charlesson B, O' Neill H, Ross ML, et al. The Effects of Dietary Pattern during Intensified Training on Stool Microbiota of Elite Race Walkers. Nutrients. 2019 Jan 24;11(2):261.

13. Petersen, Lauren M., Eddy J. Bautista, Hoan Nguyen, Blake M. Hanson, Lei Chen, Sai H. Lek, Erica Sodergren, and George M. Weinstock. "Community characteristics of the gut microbiomes of competitive cyclists." Microbiome 5, no. 1 (2017): 1-13.

14. Scheiman, Jonathan, Jacob M. Luber, Theodore A. Chavkin, Tara MacDonald, Angela Tung, Loc-Duyen Pham, Marsha C. Wibowo et al. "Meta-omics analysis of elite athletes identifies a performance-enhancing microbe that functions via lactate metabolism." Nature medicine 25, no. 7 (2019): 1104-1109.

15. Tabone M, Bressa C, García-Merino JA, Moreno-Pérez D, Van EC, Castelli FA, Fenaille F, Larrosa M. The effect of acute moderate-intensity exercise on the serum and fecal metabolomes and the gut microbiota of cross-country endurance athletes. Scientific reports. 2021 Feb 11;11(1):1-2.

16. Taniguchi H, Tanisawa K, Sun X, Kubo T, Hoshino Y, Hosokawa M, et al. Effects of short-term endurance exercise on gut microbiota in elderly men. Physiol Rep. 2018 Dec;6(23):e13935.

17. Zhao, Xia, Zhujun Zhang, Bin Hu, Wei Huang, Chao Yuan, and Lingyun Zou. "Response of gut microbiota to metabolite changes induced by endurance exercise." Frontiers in microbiology 9 (2018): 765.
